# Parameter optimization and yield prediction of cathode coating separation process for direct recycling of end-of-life lithium-ion batteries†

**DOI:** 10.1039/d1ra04086c

**Published:** 2021-07-09

**Authors:** Liurui Li, Tairan Yang, Zheng Li

**Affiliations:** Department of Mechanical Engineering, Virginia Tech Blacksburg VA 24060 USA zhengli@vt.edu

## Abstract

Fast adoption of lithium-ion batteries (LIBs) for electric vehicles requires an effective LIB recycling process to recover the valuable battery components and alleviate the concerns over the disposal of hazardous waste. The retrieval efficiency of cathode materials in direct recycling of end-of-life (EOL) lithium-ion batteries is systematically studied using the Taguchi Design of Experiment (DoE) method for the first time. A mathematical regression model is also developed to predict the yield and guide the parameter selection.

## Abstract 2

The pre-treatment efficiency of the direct recycling strategy in recovering end-of-life Li-ion batteries is predicted with levels of control factors.

## Graphical abstract

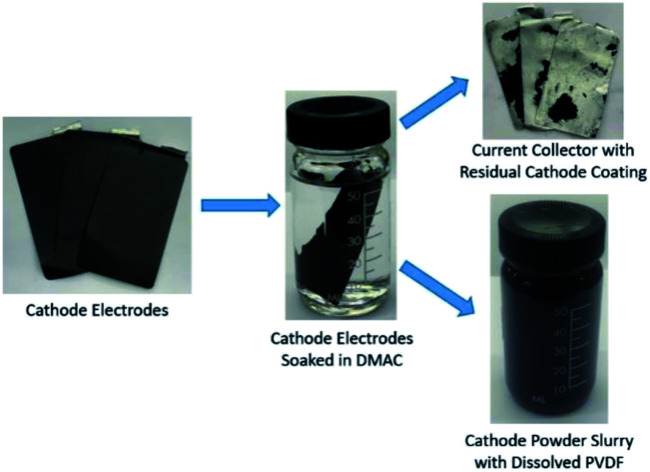

## Introduction

Recycling EOL LIBs is not only necessary but also urgently needed in recent years. EOL LIBs are metal enriched city “mine” for lithium, cobalt, manganese, and nickel. Generally 1 ton of lithium can be recycled out of 28 tons of EOL lithium ion batteries.^1,2^ It takes 250 tons of mineral ore spodumene along with 1900 tons of water to extract the same amount of lithium.^3^ Meanwhile, discarded LIBs are serious environmental hazards. Residual electrical capacity tends to cause explosions or fire accidents. Commonly used LIB electrolyte salt (*e.g.*, lithium hexafluorophosphate) reacts with water and releases harmful hydrofluoric acid vapor. In terms of urgency, the increasing demand of LIBs in EV market since 2010 (ref. 4) indicates that there will be a heavy burden on the original equipment manufacturers (OEMs) and governments in the foreseen future since merely 4 to 15 years' battery module lifetime^5,6^ is expected. At the same time, the bursting demand of LIBs will also impose great pressure on the supply chain of critical raw materials such as cobalt. The price of cobalt rose by more than 80% in 2017.^7^

Currently, there are three main recycling methods in industry: pyrometallurgical recycling (PR), hydrometallurgical recycling (HR), and direct recycling (DR).^8–10^ Both PR and HR methods break the cathode compound down to elemental constituents and selectively extract metal elements from the mix.^11^ The simplicity of the overall metallurgical process comes with high energy consumption, large waste generation, and low capability in recovering lithium and manganese,^12^ which is challenging for enterprises to make profit out of battery recycling business. In contrast, DR has the highest material recovery rate and least waste generation among all three recycling methods and has been actively developing in research labs towards industrial scale applications.^13–17^ Since the cathode morphology is well-preserved during the entire direct recycling process, the cathode materials instead of elemental constituents can be recycled and reused.^12^

Commonly used material extraction processes in LIB recycling involve some types of physical or chemical separation process, such as shredding,^18^ thermal treatment,^19^ and organic solvent methods. The shredding method, which involves multilevel crushing, fine sieving and air-classification,^20^ introduces tremendous amount of impurities that are hard to purify in subsequent processes. The thermal treatment method easily leads to change in the structure, composition, and morphology of cathode materials. In our DR, we have developed a novel pre-sorting process to separate cathode sheet, anode sheet and separator^21^ and an organic solvent extraction process to retrieve active cathode powder by dissolving the binder (*e.g.*, polyvinylidene fluoride, PVDF) *via* solvent soaking and sonication.

**Fig. 1:**
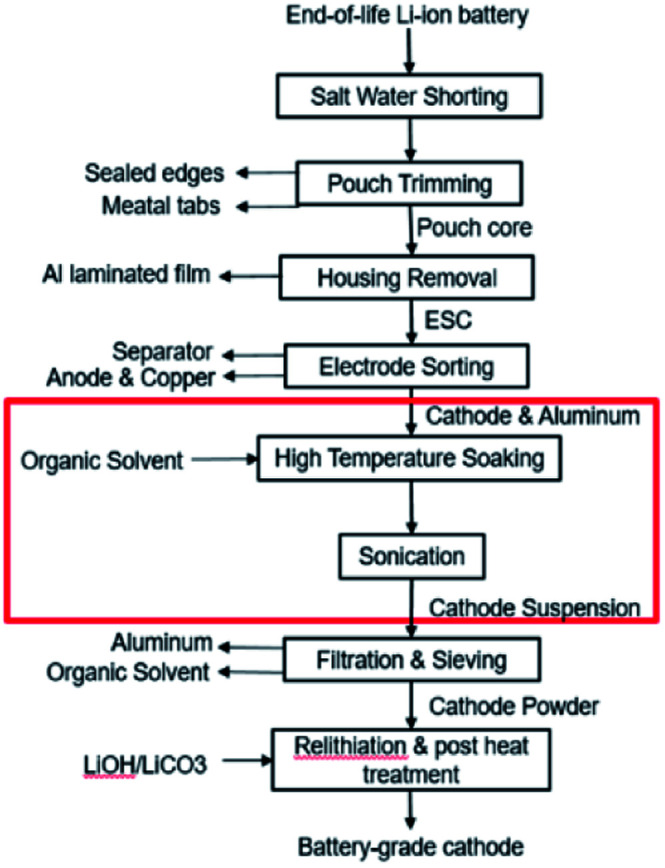
Direct recycling for recovery of cathode coating from end-of-life LIBs. Focus of this work highlighted in red.

This paper focuses on the organic solvent extraction method (Fig. 1) and studies the relationship between the processing parameters and the cathode material retrieval yield using Taguchi DoE methods and regression analysis. Processing parameters that have minor influences on the material retrieval yield from a single cathode sheet are first identified by the Placket–Burman parameter screening method and set to a level that would benefit the yield the most. The remaining parameters along with essential parameters for potential mass production process are evaluated by Taguchi DoE. Finally, the results of Taguchi DoE will be used to generate a regression model that is able to predict the yield under different input parameter combinations.

## Experimental

In this work, EOL battery cells (Fig. S1†) were randomly selected from a pool of waste drone battery modules provided by a third-party battery recycler. In order to minimize the residual energy, the battery modules were discharged by a BD200 battery discharger (Fig. S2a†) to an average cell voltage of below 2 V per battery. Discharged modules were then manually disassembled into individual cells. The voltage of each single pouch cell needs to be double-checked before being cut open for extracting the electrode-separator compound (ESC). *Z*-folded ESC structure is found on all selected modules, which results in 60 to 70 single sheet electrodes from each battery. Our previous research^21^ indicated that fully automated cathode and anode separation of *Z*-folded Li-ion batteries was feasible, thus in this work we focused on process parameters that would influence the yield of cathode powder extraction from the cathode sheets. During the organic solvent extraction process, the cathode active materials are retrieved by breaking particle-to-particle and particle-to-Al current collector bonding forces formed by binders. The most commonly found binder type is PVDF, which dissolves in organic solvents such as *N*-methyl-2-pyrrolidone (NMP), dimethylacetamide (DMAC), dimethylformamide (DMF), dimethylsulfoxide (DMSO) and acetone. X. Song^15^ indicated DMAC and DMF outperform other solvents on dissolution effectiveness and cost efficiency. Thus, DMAC and DMF were used as soaking and sonication media in our experiments. The high temperature soaking and sonication processes were conducted in a convection oven (Fig. S2b†) and ultrasonic cleaner (Fig. S2c†).

## Results and discussion

The cathode material yield during the material retrieval process is estimated by weight difference before and after the soaking–sonication process. On an average, the weight of cathode electrodes (*W*_initial_) consists of 28% Al current collector and 72% cathode coating. After the cathode separation process, residual cathode electrodes are dried in an oven and weighed (*W*_post_) again. The final yield of the cathode separation process can then be estimated by eqn (1), which is referred to as response *Y* and yield in the rest of this paper.1
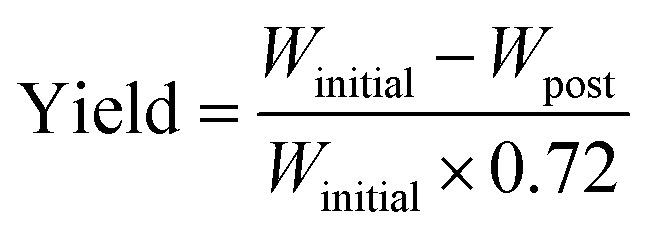


**Fig. 2:**
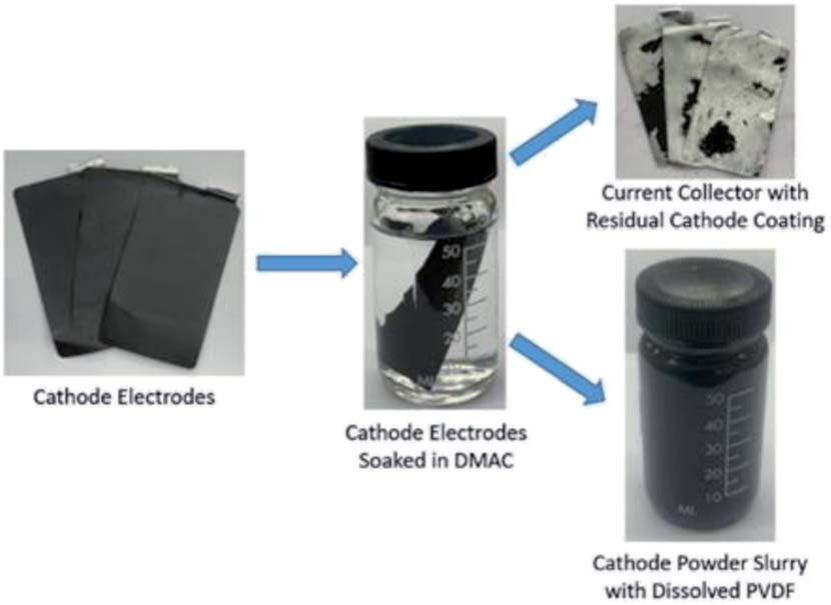


**Fig. 3:**
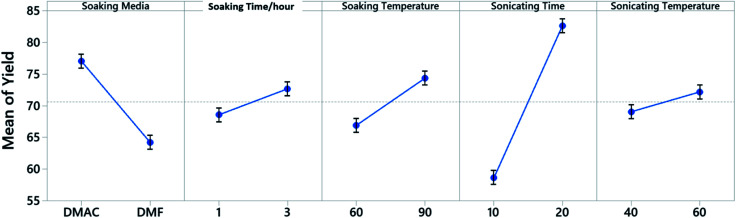
Main effects plot for fitted mean of screening experiment yield.

A 5 factor 2 level Placket–Burman screening experiment is constructed to identify the control factors that would influence the yield when dealing with a single cathode sheet (Fig. 2). The Placket–Burman experiment design ensures that each combination of levels for any pairs of factors are studied for the same number of times, similar to a complete factorial design but with a smaller number of runs. For factors and levels given in Table 1, a complete factorial design will require 32 runs while the Placket–Burman design only needs 12 runs (Table S1†). Each run is repeated 5 times with a single cathode sheet randomly picked from a shuffled electrode pool and responses are averaged and recorded in the last column of Table S1.† The averaged responses are further tested with analysis of variance (ANOVA) to determine the significance of each factor. The results constructed at 95% confidence interval from Minitab 19 are shown in Table S2.† Factors with *P*-values less than 0.05 are considered to have statistically significant contribution to the response *Y*, which is the cathode material retrieval yield in our case. Here, *P*-value is the quantified significance level that indicates how much the responses from different levels of a control factor disagree with null hypothesis. The *P*-value obtained from the *F*-distribution table at 95% confidence interval indicates that the sonication time with a *P*-value of 0.003 is a decisive factor towards the yield and will be factored in for the following Taguchi experimental design. As a quantitative factor, the soaking media have a *P*-value of 0.046 and the main effect plot (Fig. 3) shows that DMAC outperforms DMF. Therefore, DMAC was chosen to be the organic solvent media in Taguchi experimental design. Other factors in Table 1 such as soaking time, soaking temperature, and sonication temperature have *P*-values larger than 0.05 and are considered as insignificant. These insignificant factors are taken out of the factor list for Taguchi experimental design and their pre-set levels are listed in Table S3† along with other essential pre-set process parameters.

**Table 1:** Screening experiment parameters and levels

Factor	Soaking media (A), (type)	Soaking time (B), (hour)	Soaking temperature (C), (°C)	Sonication time (D), (second)	Sonication temperature (E), (°C)
Level 1	DMAC	2	60	10	40
Level 2	DMF	6	90	20	60

Studying a single electrode at a time can be done at lab-scale to better understand the relationship between processing parameters and material retrieval yield in the organic solvent extraction process. In an industry relevant environment, however, mass production requires much higher process throughput and introduces more process parameters that need to be systematically studied. Thus, to simulate the industrial material retrieval process, shuffled cathode electrodes were cut into smaller pieces of controlled size and treated at a much higher solid-to-liquid ratio in the following study.

First, we used the Taguchi DoE method to identify the significance of each of the control factors chosen in Table 2. The Taguchi DoE method is an effective statistical off-line quality control methodology aimed at increasing the robustness of a product or process facing variations over which we have minimum control in the design stage. It is capable of studying both control factors that are controllable during the production and noise factors that we cannot control when the process is in use. On top of the experimental results from Taguchi DoE, a regression analysis was developed to predict the yield and processing parameter selection. Table 2 lists all 3 factors and 4 levels to be studied in Taguchi DoE, which enabled us to develop a more precise regression model compared to the 2 level Placket–Burman parameter screening experiment. In this study, the L16(4^3^) Taguchi orthogonal array, which is a highly fractional factorial design, is applied to select subsets of combinations of 3 control factors at 4 levels. The Taguchi orthogonal arrays are well balanced to independently evaluate all levels and factors, thus each factor and level can be equally considered. The detailed subset combinations are as listed in Table S4.† Each subset combination in L16(4^3^) Taguchi experiment design is repeated 5 times and yields are averaged and recorded in the last column of Table S4.† The statistical analysis of yields is carried out using Minitab.

**Table 2:** Taguchi experiment parameters and levels

Factor	Sonication time (A), (min)	Sheet size (B), (cm^2^)	Solid–liquid weight ratio (C), (mg ml^−1^)
Level 1	1	0.52	5
Level 2	3	1.04	10
Level 3	5	2.09	15
Level 4	7	4.18	20

A continuous quality loss function is used to evaluate the performance characteristics of the Taguchi DoE method. This loss function calculates the deviation of a design parameter from the desired value. The value of this loss function is called the signal-to-noise (S/N) ratio. Three categories of S/N ratios are available depending on the goal of experiments:

If the response is to be maximized, that is the larger is better, then:2
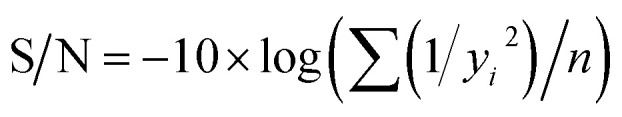


If the response is better to be an intermediate value, then:

3 S/N = 10 × log(*ȳ*^2^/*σ*^2^)

If the response is to be minimized, that is the smaller is better, then:4
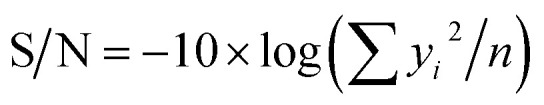
where *y*_*i*_ is the response of the *i*_th_ run, *ȳ* is the average response of all runs, *σ* is the standard deviation of the response, and *n* is the total number of runs.

Here in our case, to study the yield of cathode material retrieval, we expected the response to be the higher the better. Thus S/N ratios for yield are calculated from the first S/N calculation equation. For the next phase of the study, the aluminium impurity will be introduced as the second response. S/N ratios for this response will be calculated by “the smaller is better” principle as aluminium debris from the current collector is an unwanted element in cathode material retrieved.

**Fig. 4:**
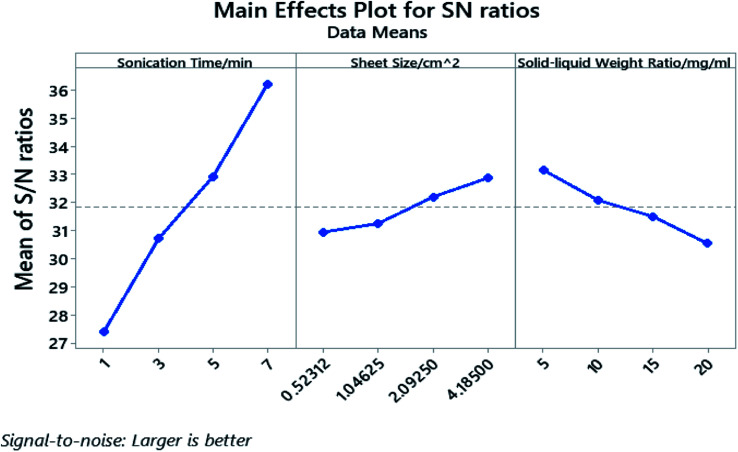
Main effects plot for S/N ratios in Taguchi experiment.

The processing parameters were ranked based on their influence on the yield as well as how these parameters influence the yield, and are shown in the response table for S/N ratio (Table 3) and main effect plot (Fig. 4). The parameters that influence the cathode yield from high to low are sonication time (A), solid–liquid weight ratio (C), and sheet size (B). Both sonication time and sheet size have positive impact on the yield as their level increases, while the solid–liquid weight ratio shows negative influence on the yield with more electrodes being added into certain amount of organic solvent.

**Table 3:** Response table (S/N ratio) for Taguchi experiment

Level	Sonication time (A)	Sheet size (B)	Solid–liquid weight ratio (C)
1	27.39	30.95	33.16
2	30.71	31.25	32.08
3	32.94	32.21	31.51
4	36.25	32.88	30.54
Delta^a^	8.86	1.94	2.61
Rank	1	3	2

a This value represents the difference between the highest and lowest S/N ratio for each factor.

To quantify the importance of each control factor and their specific contribution towards the yield, an analysis of variance of the S/N ratio was carried out with a confidence interval of 95%. This means that as long as the *P*-value of a factor shown in Table S5† is less than 0.05, this control factor can be considered to have statistically significant influence towards the cathode yield. Individual contribution (*P*%) of *i*_th_ factor towards the cathode yield is calculated by eqn (5):5
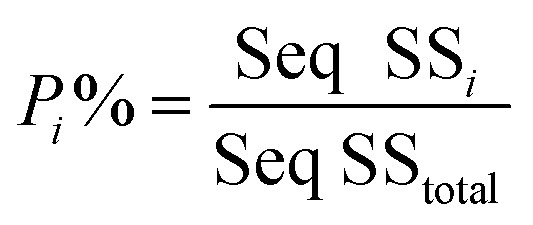
where Seq SS_*i*_ is the sequential sum of squares for *i*_th_ factor and Seq SS_total_ is the sequential sum of squares of all factors.

The *P*-value of the sonication time (A), the sheet size (B), and the solid–liquid weight ratio (C) proved to be well below 0.05 and the *P*-value rank agrees with the rank indicated by the response table for S/N ratio. The calculated individual contribution (*P*%) in Table S5† indicate that the sonication time has the highest contribution of 86.55% followed by solid–liquid weight ratio at 7.44% and sheet size at 4.90%.

As all three factors prove to be statistically significant towards the cathode yield, a linear regression model is established to correlate the cathode yield and levels of all factors by analysing results of Taguchi experiments. The model results are as follows:6
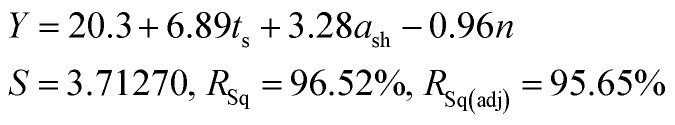
where *Y* represents the cathode yield in %, *t*_s_ represents the sonication time in min, *a*_sh_ represents the sheet size in cm^2^, and *n* represents the solid–liquid ration in mg ml^−1^. *S* represents the average distance that the observed values fall from the regression line, and *R*-square (*R*_Sq_) explains the degree to which the control (input) variables explain the variation of predicted response, adjusted *R*_Sq(adj)_ is similar to *R*_Sq_ but eliminates the influence of relatively insignificant input variables.

To validate the accuracy of the linear regression model, confirmation tests were performed to compare the predicted cathode yield and the experimental values. As shown in Table S6,† three additional runs were randomly picked from the factor combination pool of the full factorial design. The averaged error of the three validation experiments was 3.35%, which is within an acceptable range.

## Conclusion

In summary, this study has identified sonication time, sheet size, and solid–liquid weight ratio as three essential control factors towards the material retrieval yield by organic solvent extraction. DMAC outperformed DMF and proved to be a cost-effective organic solvent with the highest yield for material retrieval. The S/N ratio analysis in Taguchi DoE reveals the contribution of sonication time (86.55%), solid–liquid weight ratio (7.44%), and sheet size (4.90%) towards the final yield. The mathematical relationship between the yield and control factors are successfully established with the results of Taguchi DOE and proved accurate by confirmation tests. The success in yield prediction enables us to study the aluminium impurity introduced by the sonication process as the second response in future studies.

## Conflicts of interest

There are no conflicts to declare.

## Supplementary Material

RA-011-D1RA04086C-s001
