# Supplementary material for: Parameter optimization and yield prediction of cathode coating separation process for direct recycling of end-of-life lithium-ion batteries
Source: RSC Adv. 2021 Jul 9;11(39):24132–6. doi: 10.1039/d1ra04086c (PMC9036732; doi:10.1039/d1ra04086c)
Supplement: RA-011-D1RA04086C-s001 [file RA-011-D1RA04086C-s001.pdf]

Electronic Supplementary Material (ESI) for RSC Advances.
This journal is © The Royal Society of Chemistry 2021

COMMUNICATION

Parameter Optimization and Yield Prediction in Cathode Coating Separation Process for Direct Recycling of End-Of-Life Lithium-ion Batteries †

Liurui Li[a], Tairan Yang[a] and Zheng Li*[a]

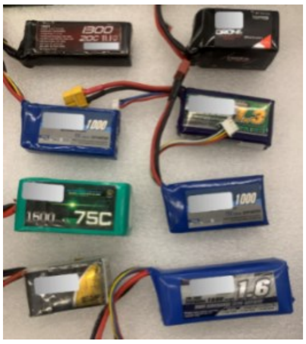

Figure S1. Battery modules disassembled for DOE

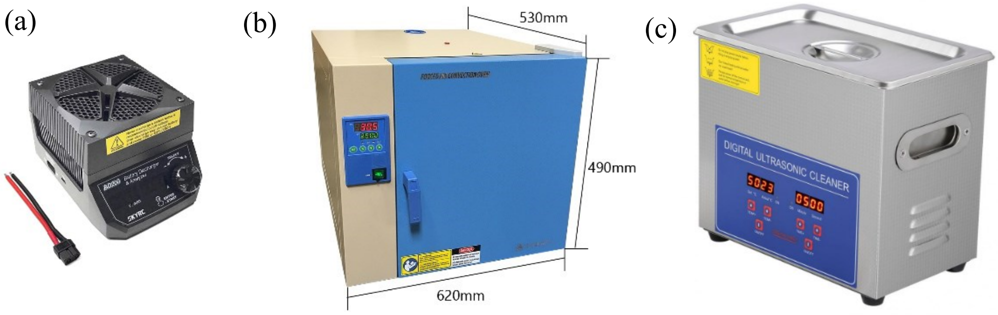

Figure S2. Equipment requirement (a) Battery module discharger, (b) High temperature oven and (c) Ultrasonic cleaner

Please do not adjust margins

Table S1. Plackett-Burman Screening Experiment Design and Result

RunOrder	Soaking Media(A)	Soaking Time hour (B)	Soaking Temperature ℃ (C)	Sonicating Time min (D)	Sonicating Temperature ℃ (E)	Yield % (Y)
1	DMAC	3	60	10	40	63.7
2	DMF	1	90	10	40	50.4
3	DMAC	3	90	10	60	70.2
4	DMF	1	60	10	60	52.6
5	DMAC	1	60	10	40	49.5
6	DMF	3	60	20	60	61.7
7	DMF	1	90	20	40	75.2
8	DMAC	3	90	20	40	95.1
9	DMF	3	90	10	60	64.9
10	DMF	3	60	20	40	80.3
11	DMAC	1	60	20	60	93.4
12	DMAC	1	90	20	60	90.2

Table S2. Analysis of Variance of Screening Experiment

Source	DF	Adj SS	Adj MS	F-Value	P-Value
A	1	494.08	494.08	6.27	0.046
B	1	50.43	50.43	0.64	0.454
C	1	167.25	167.25	2.12	0.195
D	1	1742.43	1742.43	22.12	0.003
E	1	29.45	29.45	0.37	0.563
Error	6	472.57	78.76		
Total	11	2956.22			

Table S3. Pre-set Process Parameters

Parameters	Soaking Media	Soaking Time	Soaking Temperature	Sonicating Temperature	Sonicating Frequency
Unit	NA	Hour	℃	℃	kHz
Level	DMAC	5	90	60	40

This journal is © The Royal Society of Chemistry 20xx

Please do not adjust margins

Please do not adjust margins

Table S4. Taguchi L16 (4^3) Orthogonal Array

RunOrder	Sonication time(A)	Sheet Size(B)	Solid-liquid Ratio(C)	Yield(Y)
1	1	0.52312	5	25.4
2	1	1.04625	10	23.5
3	1	2.09250	15	21.9
4	1	4.18500	20	23.0
5	3	0.52312	10	30.8
6	3	1.04625	5	36.4
7	3	2.09250	20	31.2
8	3	4.18500	15	39.7
9	5	0.52312	15	37.8
10	5	1.04625	20	34.2
11	5	2.09250	5	57.9
12	5	4.18500	10	51.7
13	7	0.52312	50	52.3
14	7	1.04625	15	60.9
15	7	2.09250	10	69.8
16	7	4.18500	5	79.9

Table S5. Taguchi Experiment Analysis of Variance for S/N Ratio

Source	DF	Seq SS	Adj SS	Adj MS	F-Value	P-Value	P% Contribution
A	3	166.807	166.807	55.6023	155.43	0.000	86.55
B	3	9.443	9.443	3.1478	8.8	0.013	4.90
C	3	14.343	14.343	4.7810	13.36	0.005	7.44
Error	6	2.146	2.146	0.3577			1.11
Total	15	192.74					100

DF: degrees of freedom; Seq SS: sequential sum of squares; Adj SS: adjusted sum of squares; Adj MS: adjusted mean squares.

Table S6. Regression Analysis Verification Tests

Please do not adjust margins

Please do not adjust margins

Experiment No.	Sonication Time	Sheet Size	Solid-liquid Ratio	Predicted Value(%)	Experiment Value(%)	Error(%)
1	3	0.523	15	28.26	26.75	5.36
2	5	1.485	10	58.58	58.99	0.22
3	7	2.092	5	70.56	73.73	4.49

This journal is © The Royal Society of Chemistry 20xx

Please do not adjust margins
